# Opposite Associations of Plasma Homoarginine and Ornithine with Arginine in Healthy Children and Adolescents

**DOI:** 10.3390/ijms141121819

**Published:** 2013-11-04

**Authors:** Aleksandra JaŸwińska-Kozuba, Jens Martens-Lobenhoffer, Olga Kruszelnicka, Jarosław Rycaj, Bernadeta Chyrchel, Andrzej Surdacki, Stefanie M. Bode-Böger

**Affiliations:** 1 Almed-Elektra Medical Center, 41-710 Ruda Œląska, Poland; E-Mail: almedelektra@interia.pl; 2 Institute for Clinical Pharmacology, Otto-von-Guericke University, 39120 Magdeburg, Germany; E-Mails: jens.martens-lobenhoffer@med.ovgu.de (J.M.-L.); stefanie.bode-boeger@med.ovgu.de (S.M.B.-B.); 3 Department of Coronary Artery Disease, the John Paul II Hospital, 31-202 Cracow, Poland; E-Mail: olga.kruszelnicka@onet.pl; 4 Department of Cardiology, Congenital Heart Defects and Electrotherapy, Silesian Center for Heart Diseases in Zabrze, Medical University of Silesia, 40-055 Katowice, Poland; E-Mail: jacyr@interia.pl; 5 2nd Department of Cardiology, Jagiellonian University Medical College and University Hospital, 31-501 Cracow, Poland; E-Mail: bernadkawitanek@poczta.onet.pl

**Keywords:** homoarginine, ornithine, arginine, children, carotid vascular structure

## Abstract

Homoarginine, a non-proteinogenic amino acid, is formed when lysine replaces ornithine in reactions catalyzed by hepatic urea cycle enzymes or lysine substitutes for glycine as a substrate of renal arginine:glycine amidinotransferase. Decreased circulating homoarginine and elevated ornithine, a downstream product of arginase, predict adverse cardiovascular outcome. Our aim was to investigate correlates of plasma homoarginine and ornithine and their relations with carotid vascular structure in 40 healthy children and adolescents aged 3–18 years without coexistent diseases or subclinical carotid atherosclerosis. Homoarginine, ornithine, arginine, asymmetric dimethylarginine (ADMA) and symmetric dimethylarginine (SDMA) were measured by liquid chromatography-tandem mass spectrometry with stable isotope-labeled internal standards. Intima-media thickness (IMT) and extra-medial thickness (EMT) of common carotid arteries were estimated by B-mode ultrasound. Homoarginine correlated with arginine (*r* = 0.43, *p* = 0.005), age (*r* = 0.42, *p* = 0.007) and, weakly, with an increased arginine-to-ornithine ratio, a putative measure of lower arginase activity (*r* = 0.31, *p* = 0.048). Ornithine correlated inversely with arginine (*r* = −0.64, *p* < 0.001). IMT, EMT or their sum were unrelated to any of the biochemical parameters (*p* > 0.12). Thus, opposite associations of plasma homoarginine and ornithine with arginine may partially result from possible involvement of arginase, an enzyme controlling homoarginine degradation and ornithine synthesis from arginine. Age-dependency of homoarginine levels can reflect developmental changes in homoarginine metabolism. However, neither homoarginine nor ornithine appears to be associated with carotid vascular structure in healthy children and adolescents.

## 1. Introduction

Homoarginine is a non-proteinogenic, basic amino acid that differs from arginine by an additional methylene group in the carbon chain. Homoarginine is formed when lysine replaces ornithine in reactions catalyzed by hepatic enzymes of the urea cycle [1–4] or lysine substitutes for glycine as a substrate of renal arginine:glycine amidinotransferase (AGAT) [1,5–7]. Decreased levels of circulating homoarginine have recently emerged as an independent predictor of all-cause and cardiovascular mortality in 3305 Ludwigshafen Risk and Cardiovascular Health (LURIC) study participants referred for coronary angiography and in 1244 diabetic patients on maintenance hemodialysis from the 4D (Die Deutsche Diabetes Dialyse) study [8]. In addition, transient elevations of homoarginine, but not simultaneous changes in arginine or asymmetric dimethylarginine (ADMA), an endogenous nitric oxide (NO) synthase inhibitor, were related to enhanced endothelial function during the second and third trimesters of pregnancy [9,10]. The ability of homoarginine to modulate NO bioavailability in the presence of about 30-fold higher arginine concentrations [8] appears as intriguing as the arginine paradox, *i.e.*, the capability of exogenous arginine to stimulate NO formation in spite of a much lower Michaelis-Menten constant (*K*_m_) of endothelial-type NO synthase (eNOS) for arginine compared to circulating arginine levels. Assuming an effect of homoarginine on NO bioavailability and, consequently, early atherogenesis, an association of homoarginine with carotid vascular structure may be hypothesized.

Additionally, the LURIC study identified an independent association of cardiovascular mortality with higher concentrations of ornithine, a product of arginase activity [11]. However, to the best of our knowledge, relations of homoarginine or ornithine with carotid intima-media thickness (IMT) have not been reported so far.

It has long been recognized that homoarginine levels are decreased in patients with renal dysfunction [8,12], in accordance with the notion of the importance of renal AGAT for homoarginine formation [7]. Effects of age on blood homoarginine are less clear. Although März *et al.* [8] described a weak negative correlation between homoarginine and age in the LURIC cohort (mean age, 63 ± 11 years), Meinitzer *et al.* [13] have not observed any association of homoarginine and age in healthy adult men aged 20–75 years.

To the best of our knowledge, determinants of homoarginine or ornithine levels in healthy children have not been reported so far. Therefore, our aim was to estimate correlates of plasma homoarginine and ornithine and their association with carotid vascular structure in healthy subjects below 18 years of age. Beyond IMT, we also investigated relations between homoarginine, ornithine and carotid extra-medial thickness (EMT)—a recently proposed measure providing insight into adventitial remodeling [14,15], implicated in early atherogenesis [16–19]. As EMT had been demonstrated to be linked to modifiable cardiovascular risk factors more closely than IMT [14], we hypothesized that associations of EMT with homoarginine or ornithine might be revealed in a group of healthy children without coexistent diseases or subclinical carotid atherosclerosis, selected on the basis of a wide set of exclusion criteria.

## 2. Results

Biochemical characteristics of our study group have been presented in Table 1, as previously described [20]. Median plasma homoarginine concentration was 1.41 μmol/L (interquartile range, 1.14–1.82 μmol/L), being skewed to the right, as reported previously in a cohort of 136 healthy adults [21]. Arginine and ornithine levels averaged 69 ± 22 (mean ± SD) μmol/L and 72 ± 32 μmol/L, respectively, and the mean arginine-to-ornithine ratio was 1.24 ± 0.79. Respective mean levels of asymmetric dimethylarginine (ADMA) and symmetric dimethylarginine (SDMA) were 0.63 ± 0.12 μmol/L and 0.56 ± 0.10 μmol/L [20]. Median values of averaged IMT and EMT were 0.45 mm (0.41–0.53 mm) [20] and 0.65 mm (0.56–0.78 mm), respectively.

**Figure 1:**
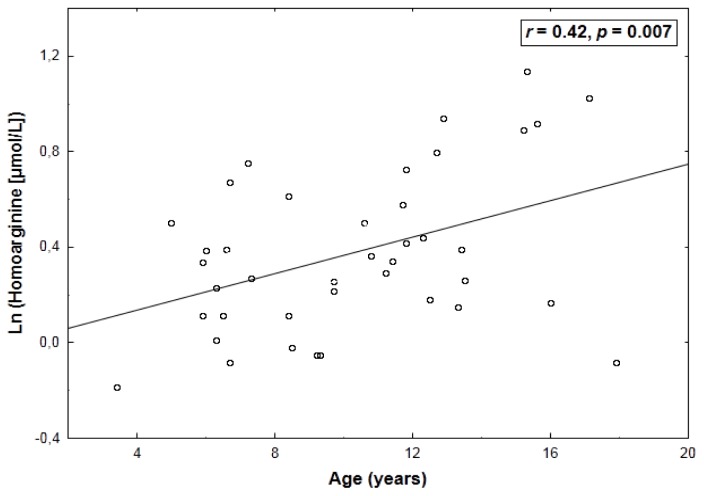
A positive correlation between age and natural logarithmically (ln)-transformed plasma levels of homoarginine.

Natural logarithmically (ln)-transformed concentrations of homoarginine correlated positively with arginine (*r* = 0.43, *p* = 0.005) (Table 2) and age (*r* = 0.42, *p* = 0.007) (Figure 1). Additionally, a weak—albeit significant—relationship of homoarginine with an increased arginine-to-ornithine ratio was found (*r* = 0.31, *p* = 0.048). By multiple linear regression, ln-transformed homoarginine was associated with arginine (β = 0.42 ± 0.13, *p* = 0.003) and age (β = 0.40 ± 0.13, *p* = 0.005) (adjusted *R*^2^: 0.31, *p* < 0.001).

**Figure 2:**
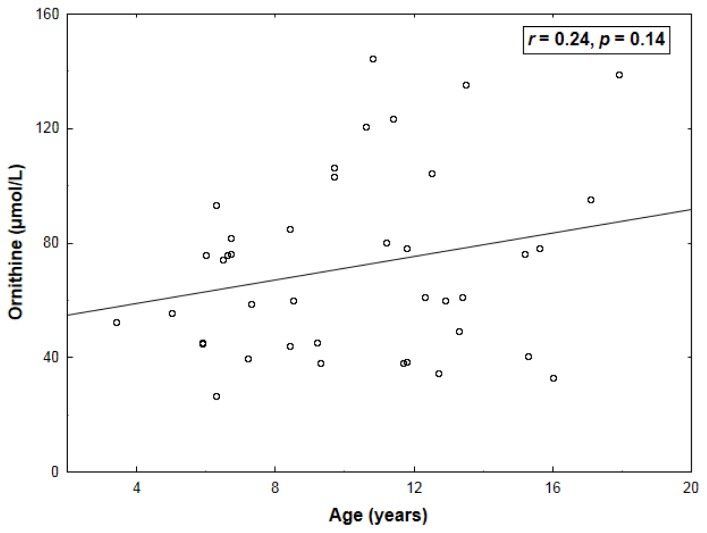
Lack of correlation between age and plasma ornithine levels.

Ornithine correlated inversely with arginine (*r* = −0.64, *p* < 0.001) and glucose (*r* = −0.43, *p* = 0.006) (Table 2) and tended to increase insignificantly with age (*r* = 0.24, *p* = 0.14) (Figure 2). By a multivariate approach, arginine (β = −0.56 ± 0.14, *p <* 0.001) was the only ornithine predictor (adjusted *R*^2^: 0.39, *p* < 0.001). Both homoarginine and ornithine were unrelated to ADMA or SDMA (*p* > 0.15).

Neither ln (IMT) nor ln (EMT) nor ln (IMT + EMT) correlated to homoarginine, ornithine, ADMA, SDMA, l-arginine or the arginine-to-ornithine ratio (*p* > 0.12).

## 3. Discussion

### 3.1. Comparison with Other Reports on Circulating Homoarginine and Ornithine

In our healthy subjects below 18 years of age, plasma homoarginine levels were lower by almost 50% compared to homoarginine concentrations (≈2.5 μmol/L) previously reported in 292 healthy non-smoking 20–75-year-old men [13] and 136 apparently healthy volunteers aged 48 ± 11 years [21]. In addition, serum homoarginine was intermediate (≈2.0 μmol/L) in 68 healthy juveniles aged 8–21 years (mean, 13.2 years, *i.e.*, higher by three years compared to our study group) [22]; nevertheless, correlates of homoarginine were not determined in that report. Accordingly, an increase of homoarginine with age in our healthy children and adolescents might result from gradual maturation of the pathways controlling homoarginine metabolism. An age-dependency of plasma concentrations of several amino acids had previously been described in a healthy pediatric population [23,24], nevertheless, homoarginine was not measured in those studies. Additionally, in contrast to homoarginine, plasma arginine was independent of age in our study group, in agreement with a previous report on 54 healthy children with a mean age of 11.9 ± 4.6 years [25].

With regard to ornithine, in our subjects, the levels of this amino acid were about 50% higher compared to those previously described in healthy children of a similar age [23,24]. A weak increase in plasma ornithine between six and sixteen years of age was reported by Lepage *et al.* [23], which was confirmed in our study.

### 3.2. Proposed Mechanisms of the Relations between Homoarginine, Arginine and Ornithine

That in the present study plasma homoarginine correlated positively with arginine—in agreement with a report on the LURIC cohort [8]—is consistent with the notion of the predominant role of the promiscuous activity of renal AGAT for homoarginine formation [5–7], because AGAT transfers the amidino group from arginine to lysine instead of glycine. The importance of this pathway for homoarginine synthesis has been suggested on the basis of an increased, not decreased, homoarginine level in a patient with a deficiency of argininosuccinate synthase, a urea cycle enzyme involved in hepatic homoarginine generation [7]. Given the importance of the intestinal-renal axis in the endogenous formation of systemic arginine (separated from the hepatic arginine pool) [26,27], arginine synthesis from the gut-derived citrulline in the proximal convoluted tubule coincides in location with the site of the expression of AGAT [28]. As AGAT expression is upregulated by growth hormone, thyroxin [29] and testosterone [30], these could have contributed to the age-dependency of plasma homoarginine. Nevertheless, the lack of hormonal assays inevitably constrains speculations on the mechanisms underlying our findings.

Keeping in mind the relevance of renal AGAT for homoarginine formation and decreased homoarginine concentrations in even mild renal insufficiency despite homoarginine being excreted in the urine [12], we expected an association of homoarginine with renal function. However, neither estimated glomerular filtration rate (eGFR) nor SDMA, a measure previously shown to inversely correlate with eGFR, also in our study group [20], was related to homoarginine levels in the present study.

In the LURIC study participants, März *et al.* [8] described a relationship between homoarginine and an increased arginine-to-ornithine ratio, a putative indirect measure of lower arginase activity [8]. In our hands, homoarginine was also positively related to the arginine-to-ornithine ratio, with an *r-*value similar to the LURIC cohort [8], which could be attributable [8] to the ability of homoarginine to inhibit arginases [31], governing the predominant pathway of arginine catabolism [27]. Nevertheless, compared to lysine and ornithine, homoarginine is a much weaker arginase inhibitor [31–33], and its plasma concentrations are about 20–80-fold lower [8]. Therefore, as homoarginine can be converted by arginase to lysine and urea [2,7], we can hypothesize that higher circulating homoarginine might rather be a consequence, not a cause, of reduced arginase activity, irrespective of the mechanism of arginase modulation. An altered activity of arginases might have also affected ornithine concentrations, because plasma levels of ornithine, a product of arginase, were inversely associated with arginine, a substrate of the enzyme. Accordingly, a lower activity of arginase could increase arginine availability, decrease ornithine synthesis and increase homoarginine levels, the latter both directly, via reduced homoarginine degradation, and indirectly, via enhanced homoarginine formation from lysine and arginine by AGAT. This hypothesis may explain the opposite associations of homoarginine and ornithine with arginine in our study group.

### 3.3. Homoarginine and Ornithine *versus* the l-Arginine—NO Pathway and Carotid Vascular Structure

Santhanam *et al.* [34] elegantly demonstrated that—despite a much lower *K*_m_ of eNOS (2.9 μmol/L) [35] than arginase (1–20 mmol/L) for arginine—endothelial arginase can effectively compete with eNOS for the arginine substrate, due to high intracellular arginine concentrations in endothelial cells (about 800 μmol/L) [36–38] and a 1.5-fold higher *V*_max_ of arginase compared to eNOS. As endothelial arginase is overexpressed and upregulated in aging blood vessels [39], the notion of arginase activity as a negative modulator of plasma homoarginine might explain a considerably higher ability of decreased homoarginine than decreased arginine to predict all-cause mortality in the LURIC study [8]. Admittedly, homoarginine by itself may replace arginine as a substrate for all three isoforms of NO synthase [40–42]. On the other hand, the *K*_m_ value of neuronal-type (nNOS) and inducible-type NO synthase (iNOS) for homoarginine is higher compared to arginine (nNOS: 174 *vs.* 6 μmol/L [40], 23 *vs.* 2.7 μmol/L [42]; iNOS: 33 *vs.* 13 μmol/L [42]), and the relative activity of eNOS in the presence of homoarginine averages 25% with reference to arginine [41]. Thus, low plasma levels of homoarginine are unlikely to directly affect NO formation in the presence of abundant arginine (≈2.5 *vs.* ≈85 μmol/L [13]). Furthermore, Davids and Teerlink [43] recently demonstrated similar concentrations of homoarginine in peripheral blood mononuclear cells compared to plasma (2.4 *vs.* 2.0 μmol/L), whereas intracellular arginine was over seven-fold higher (717 *vs.* 98 μmol/L), which further increased the intracellular ratio of arginine to homoarginine.

Therefore, it has been proposed that homoarginine might affect cardiovascular risk by mechanisms other than being a simple NO precursor [8], and the previously discussed hypothetical associations of homoarginine with the activity of endothelial arginases appear a plausible possibility, all the more because the role of arginases in endothelial dysfunction and atherogenesis, as well as increased vascular stiffness appears well established [34,44]. A variety of pro-atherosclerotic factors increase the expression/activity of arginases in endothelial cells [44], and upregulation of endothelial type II arginase has been implicated in atherogenesis in apolipoprotein E-deficient mice [45]. Additionally, arginase inhibition prevented eNOS uncoupling, corrected endothelial dysfunction and improved aortic compliance in aged animals [39,46,47]. Moreover, arginase blockade protected cultured endothelial cells from accelerated senescence [48]. Finally, in a recent clinical study, arginase inhibition improved endothelial function in patients with coronary artery disease and type 2 diabetes, but not in matched controls [49]. Thus, hypothetical relations with high activity of arginases might possibly contribute to the association of low homoarginine levels with adverse cardiovascular outcome.

On the other hand, we have observed no associations between homoarginine and either carotid IMT, EMT or their sum, which could be expected assuming a relevance of homoarginine for early carotid remodeling. In agreement with this hypothesis, low homoarginine might result from an increased activity of arginases that compete with eNOS for the common substrate, arginine [44]. Furthermore, ornithine, a downstream product of arginase, is a precursor of proline and polyamines that enhance collagen synthesis and vascular smooth muscle proliferation, respectively, both of which are involved in early arterial remodeling [34,44]. In 2236 patients from the LURIC study, Sourij *et al.* [11] observed that higher ornithine by itself, but not altered arginine or citrulline, was responsible for independent associations of cardiovascular mortality with a lower arginine-to-ornithine ratio or so-called “global arginine bioavailability ratio”, calculated as arginine divided by the sum of citrulline and ornithine. Moreover, in 1001 subjects participating in the Étude du Vieillissement Artériel (EVA) study, Dumont *et al.* [50] identified the relationship of a four-year increase in carotid IMT with a polymorphism in the gene of ornithine decarboxylase antizyme-1 that inhibits and accelerates the degradation of ornithine decarboxylase, a key enzyme in polyamine synthesis.

Accordingly, as IMT and EMT include arterial media and adventitia, respectively, we expected a relationship between these indices (and, especially, their sum) and homoarginine or ornithine, novel cardiovascular outcome predictors. Nevertheless, in our hands, neither homoarginine nor ornithine nor the arginine-to-ornithine ratio contributed to the variability of IMT or EMT in healthy children. Contrary to our working hypothesis, the expected associations were not revealed in children free of the majority of classical risk factors previously associated with IMT or EMT in adults [14]. Therefore, further investigations of the relations of homoarginine and ornithine with carotid vascular structure are warranted.

### 3.4. Study Limitations

First, the small number of the subjects is the major limitation of the study. Nevertheless, we made every effort to recruit exclusively healthy children and adolescents by means of a wide range of exclusion criteria, including preclinical carotid atherosclerosis [20]. Second, we have not measured serum alkaline phosphatase levels, although changes in protein turnover in children have been proposed as the basis of age-dependent decline in ADMA, correlating also positively to alkaline phosphatase [22], considered a biomarker of bone growth rate. Moreover, the ability of homoarginine to inhibit alkaline phosphatases might have contributed to associations of homoarginine with bone density and metabolism in elderly women [51]. Third, we have not estimated neither arginase activity nor any index of NO formation, which constrains mechanistic considerations based on our findings with regard to homoarginine metabolism and NO bioavailability.

## 4. Experimental Section

### 4.1. Subjects

We studied the previously described group of 40 children and adolescents (33 boys and 7 girls; mean age, 10.1 ± 3.6 years; range, 3.4–17.9 years) [20]. As reported before, exclusion criteria included congenital heart or pulmonary defects, clinical or biochemical evidence of renal or hepatic pathology, hypertension, diabetes, obesity and any other significant chronic coexistent diseases, acute disorders or relevant abnormalities in routine blood or urine analyses, considerably elevated *C*-reactive protein, as well as ultrasound evidence of atherosclerotic plaques in carotid arteries [20]. In accordance with the Helsinki Declaration, the protocol had been approved by the ethics committee of the Medical University of Silesia (No. KNW-6501-28/08), and written informed consent was obtained from the parents of each participant.

### 4.2. Biochemical Assays

Venous blood samples were drawn after an overnight fast and centrifuged, and portions of serum and plasma (collected from ethylenediaminetetraacetic acid-anticoagulated blood) were separated and frozen initially at −20 °C and, then, at −70 °C, until assayed [20]. In addition to lipids and glucose—measured by standard methods—*C*-reactive protein, homocysteine and creatinine were assessed by immunoturbidimetry (Roche Diagnostics, Basel, Switzerland), a chemiluminescent microparticle immunoassay (Abbott Diagnostics, Abbott Park City, IL, USA) and the Jaffe method with the isotope dilution mass spectrometry (IDMS)-traceable calibration (Roche Hitachi Chemistry Analyzer, Roche Diagnostics), respectively [20]. As previously described [20], an eGFR was calculated from serum creatinine and height by the revised bedside Schwartz equation [52], a formula which has been validated also for children and adolescents with normal renal function [53].

The quantification of homoarginine in plasma was performed by high performance liquid chromatography-tandem mass spectrometry (LC-MS/MS). The overall procedure of sample preparation and chromatography/detection followed the previously described method for the quantification of arginine, ADMA and SDMA [54]. Owing to the very selective and sensitive MS detection, no interferences from endogenous substances were observed. Stable isotope-labeled ^13^C_6_-arginine was used as an internal standard for endogenous homoarginine. The calibration function was linear in the range of 0.2–10 μmol/L (*R*^2^ = 1.000). The precision and accuracy of the quality control samples were better than 3% at all concentrations studied. Details of the LC-MS/MS measurements of arginine, ADMA and SDMA in the same plasma samples were described previously with ^13^C_6_-arginine and ^2^H_6_-ADMA as internal standards for arginine and both ADMA and SDMA, respectively [20,55]. Ornithine was assayed by LC-MS/MS with ^2^H_6_-ornithine as an internal standard and intra-day and inter-day relative standard deviations of 1.1% and 3.5%, respectively [56].

### 4.3. Carotid Ultrasound

As described previously [20], the common carotid artery, carotid bulb and internal carotid artery were visualized on both sides in the longitudinal plane using a high-resolution ultrasound device (iU22 xMATRIX Ultrasound System, Philips Healthcare, Best, The Netherlands) equipped with a 12-MHz linear digital ultrasound by an investigator (J. Rycaj), who was blinded to biochemical data. Beyond exclusion of atherosclerotic plaques, images were captured and stored for off-line analysis of carotid IMT and EMT. IMT was estimated within a 1 cm segment immediately proximal to the carotid bifurcation on the far wall (distal to the skin) of the common carotid artery and corresponded to the distance between the lumen-intima interface and media-adventitia interface [20,57]. Following Skilton *et al.* [14], EMT was measured along a distinct 0.3–1.0 cm long arterial segment 1–1.5 cm proximal to the carotid bulb, where the distance between the carotid artery and jugular vein is smallest. For EMT measurements, images were focused on the arterial near wall (proximal to the skin) and the venous far wall. EMT was defined as the distance between the arterial media-adventitia interface and venous lumen-intima margin, thus consisting of arterial adventitia, interstitial tissue and the entire venous wall [14,15]. The final values of IMT and EMT were averaged from 3 end-diastolic measurements per each side. We also computed a combined measure as a sum of IMT and EMT, because this parameter had previously exhibited stronger correlations with some risk factors compared to IMT or EMT [14].

### 4.4. Statistical Analysis

Continuous data are presented as means ± SD or medians and interquartile range for not normally distributed values. The accordance with a normal distribution was confirmed by Lilliefors’ test, and ln-transformation was applied when necessary for skewed variables (homoarginine, high-density lipoproteins (HDL) cholesterol, triglycerides, IMT, EMT). Relations between variables were represented by Pearson’s correlation coefficients (*r*). Independent determinants of plasma homoarginine or ornithine were assessed by multiple linear regression, including only covariates for which the *p-*value at a univariate analysis did not exceed 0.10.

Our study design allowed us to detect bivariate correlations within the study group as a whole at an *r*-value of 0.42 with a statistical power of 80% at a type I error rate of 0.05.

A *p*-value below 0.05 was inferred to be significant. Statistical tests were performed using STATISTICA (data analysis software system, version 10.0.1011.0; StatSoft, Inc., Tulsa, OK, USA).

## 5. Conclusions

Opposite associations of plasma homoarginine and ornithine with arginine may partially result from possible involvement of arginase, an enzyme controlling both homoarginine degradation and ornithine formation from arginine. The age-dependency of homoarginine levels can reflect developmental changes in pathways governing homoarginine metabolism. However, neither homoarginine nor ornithine appears to be associated with carotid IMT or EMT in healthy children and adolescents. Whether homoarginine or ornithine can be related to carotid vascular structure in other clinical settings remains to be elucidated.

## Figures and Tables

**Table 1:** Biochemical characteristics of the study subjects [20].

Variable	
Estimated GFR (mL/min per 1.73 m^2^)	122 ± 22
LDL cholesterol (mmol/L)	2.3 ± 0.6
HDL cholesterol (mmol/L)	1.5 (1.3–1.8)
Triglycerides (mmol/L)	0.7 (0.5–0.9)
Glucose (mmol/L)	4.7 ± 0.5
Homocysteine (μmol/L)	8.9 ± 2.5

Data are depicted as the mean ± SD or median (interquartile range). GFR: glomerular filtration rate; LDL: low-density lipoproteins; HDL: high-density lipoproteins.

**Table 2:** Correlation coefficients (*r*) between ornithine or homoarginine and biochemical data. ADMA, asymmetric dimethylarginine; SDMA, symmetric dimethylarginine.

	Ln-homoarginine	Ornithine
Estimated GFR	−0.11 (0.50)	−0.14 (0.39)
LDL cholesterol	−0.12 (0.45)	−0.06 (0.70)
HDL cholesterol	0.11 (0.52)	0.22 (0.18)
Triglycerides	−0.24 (0.14)	−0.07 (0.65)
Glucose	0.25 (0.12)	−0.43 (0.006)
Homocysteine	0.27 (0.10)	0.09 (0.56)
Arginine	0.43 (0.005)	−0.64 (<0.001)
ADMA	−0.05 (0.77)	0.10 (0.53)
SDMA	0.23 (0.15)	0.09 (0.56)

Respective *p*-values have been shown in parentheses. Abbreviations are as in Table 1.

## References

[1] Ryan W.L., Wells I.C. (1964). Homocitrulline and homoarginine synthesis from lysine. Science.

[2] Ryan W.L., Barak A.J., Johnson R.J. (1968). Lysine, homocitrulline, and homoarginine metabolism by the isolated perfused rat liver. Arch. Biochem. Biophys.

[3] Cathelineau L., Saudubray J.M., Charpentier C., Polonovski C. (1974). Letter: The presence of the homoanalogues of substrates of the urea cycle in the presence of argininosuccinate synthetase deficiency. Pediatr. Res.

[4] Levin B., Oberholzer V.G., Palmer T. (1974). Letter: The high levels of lysine, homocitrulline, and homoarginine found in argininosuccinate synthetase deficiency. Pediatr. Res.

[5] Ryan W.L., Johnson R.J., Dimari S. (1969). Homoarginine synthesis by rat kidney. Arch. Biochem. Biophys.

[6] Hernández-Guzmán G., Alvarez-Morales A. (2001). Isolation and characterization of the gene coding for the amidinotransferase involved in the biosynthesis of phaseolotoxin in *Pseudomonas syringae* pv. phaseolicola. Mol. Plant. Microbe Interact.

[7] Davids M., Ndika J.D., Salomons G.S., Blom H.J., Teerlink T. (2012). Promiscuous activity of arginine:glycine amidinotransferase is responsible for the synthesis of the novel cardiovascular risk factor homoarginine. FEBS Lett.

[8] März W., Meinitzer A., Drechsler C., Pilz S., Krane V., Kleber M.E., Fischer J., Winkelmann B.R., Böhm B.O., Ritz E. (2010). Homoarginine, cardiovascular risk, and mortality. Circulation.

[9] Valtonen P., Laitinen T., Lyyra-Laitinen T., Raitakari O.T., Juonala M., Viikari J.S., Heiskanen N., Vanninen E., Punnonen K., Heinonen S. (2008). Serum l-homoarginine concentration is elevated during normal pregnancy and is related to flow-mediated vasodilatation. Circ. J.

[10] Saarelainen H., Valtonen P., Punnonen K., Laitinen T., Raitakari O.T., Juonala M., Heiskanen N., Lyyra-Laitinen T., Viikari J.S., Vanninen E. (2008). Subtle changes in ADMA and l-arginine concentrations in normal pregnancies are unlikely to account for pregnancy-related increased flow-mediated dilatation. Clin. Physiol. Funct. Imaging.

[11] Sourij H., Meinitzer A., Pilz S., Grammer T.B., Winkelmann B.R., Boehm B.O., März W. (2011). Arginine bioavailability ratios are associated with cardiovascular mortality in patients referred to coronary angiography. Atherosclerosis.

[12] Marescau B., Nagels G., Possemiers I., de Broe M.E., Becaus I., Billiouw J.M., Lornoy W., de Deyn P.P. (1997). Guanidino compounds in serum and urine of nondialyzed patients with chronic renal insufficiency. Metabolism.

[13] Meinitzer A., Puchinger M., Winklhofer-Roob B.M., Rock E., Ribalta J., Roob J.M., Sundl I., Halwachs-Baumann G., März W. (2007). Reference values for plasma concentrations of asymmetrical dimethylarginine (ADMA) and other arginine metabolites in men after validation of a chromatographic method. Clin. Chim. Acta.

[14] Skilton M.R., Serusclat A., Sethu A.H.A.U., Brun S., Bernard S., Balkau B., Moulin P., Bonnet F. (2009). Noninvasive measurement of carotid extra-media thickness: Associations with cardiovascular risk factors and intima-media thickness. JACC Cardiovasc. Imaging.

[15] Skilton M.R., Sullivan T.R., Ayer J.G., Harmer J.A., Toelle B.G., Webb K., Marks G.B., Celermajer D.S. (2012). Carotid extra-medial thickness in childhood: Early life effects on the arterial adventitia. Atherosclerosis.

[16] Heistad D.D., Armstrong M.L., Marcus M.L. (1981). Hyperemia of the aortic wall in atherosclerotic monkeys. Circ. Res.

[17] Kwon H.M., Sangiorgi G., Ritman E.L., McKenna C., Holmes D.R., Schwartz R.S., Lerman A. (1998). Enhanced coronary vasa vasorum neovascularization in experimental hypercholesterolemia. J. Clin. Invest.

[18] Herrmann J., Samee S., Chade A., Rodriguez Porcel M., Lerman L.O., Lerman A. (2005). Differential effect of experimental hypertension and hypercholesterolemia on adventitial remodeling. Arterioscler. Thromb. Vasc. Biol.

[19] Michel J.B., Thaunat O., Houard X., Meilhac O., Caligiuri G., Nicoletti A. (2007). Topological determinants and consequences of adventitial responses to arterial wall injury. Arterioscler. Thromb. Vasc. Biol.

[20] JaŸwińska-Kozuba A., Martens-Lobenhoffer J., Surdacki A., Kruszelnicka O., Rycaj J., Godula-Stuglik U., Bode-Böger S.M. (2012). Associations between endogenous dimethylarginines and renal function in healthy children and adolescents. Int. J. Mol. Sci.

[21] Atzler D., Mieth M., Maas R., Böger R.H., Schwedhelm E. (2011). Stable isotope dilution assay for liquid chromatography-tandem mass spectrometric determination of l-homoarginine in human plasma. J. Chromatogr. B Analyt. Technol. Biomed. Life Sci.

[22] Gruber H.J., Mayer C., Meinitzer A., Almer G., Horejsi R., Möller R., Pilz S., März W., Gasser R., Truschnig-Wilders M. (2008). Asymmetric dimethylarginine (ADMA) is tightly correlated with growth in juveniles without correlations to obesity related disorders. Exp. Clin. Endocrinol. Diabetes.

[23] Lepage N., McDonald N., Dallaire L., Lambert M. (1997). Age-specific distribution of plasma amino acid concentrations in a healthy pediatric population. Clin. Chem.

[24] Hammarqvist F., Angsten G., Meurling S., Andersson K., Wernerman J. (2010). Age-related changes of muscle and plasma amino acids in healthy children. Amino Acids.

[25] Chobanyan-Jürgens K., Fuchs A.J., Tsikas D., Kanzelmeyer N., Das A.M., Illsinger S., Vaske B., Jordan J., Lücke T. (2012). Increased asymmetric dimethylarginine (ADMA) dimethylaminohydrolase (DDAH) activity in childhood hypercholesterolemia type II. Amino Acids.

[26] Böger R.H., Bode-Böger S.M. (2001). The clinical pharmacology of l-arginine. Annu. Rev. Pharmacol. Toxicol.

[27] Wu G., Bazer F.W., Davis T.A., Kim S.W., Li P., Marc Rhoads J., Carey Satterfield M., Smith S.B., Spencer T.E., Yin Y. (2009). Arginine metabolism and nutrition in growth, health and disease. Amino Acids.

[28] Wyss M., Kaddurah-Daouk R. (2000). Creatine and creatinine metabolism. Physiol. Rev.

[29] McGuire D.M., Tormanen C.D., Segal I.S., Van Pilsum J.F. (1980). The effect of growth hormone and thyroxine on the amount of l-arginine:glycine amidinotransferase in kidneys of hypophysectomized rats. Purification and some properties of rat kidney transamidinase. J. Biol. Chem.

[30] Hoberman H.D., Sims E.A., Engstrom W.W. (1948). The effect of methyltestosterone on the rate of synthesis of creatine. J. Biol. Chem.

[31] Hrabák A., Bajor T., Temesi A. (1994). Comparison of substrate and inhibitor specificity of arginase and nitric oxide (NO) synthase for arginine analogues and related compounds in murine and rat macrophages. Biochem. Biophys. Res. Commun.

[32] Cittadini D., Pietropaolo C., Decristofaro D., D’Ayjello Caracciolo M. (1964). *In vivo* effect of l-lysine on rat liver arginase. Nature.

[33] Fuentes J.M., Campo M.L., Soler G. (1994). Kinetics and inhibition by some aminoacids of lactating rat mammary gland arginase. Arch. Int. Physiol. Biochim. Biophys.

[34] Santhanam L., Christianson D.W., Nyhan D., Berkowitz D.E. (2008). Arginase and vascular aging. J. Appl. Physiol.

[35] Pollock J.S., Förstermann U., Mitchell J.A., Warner T.D., Schmidt H.H.H.W., Nakane M., Murad F. (1991). Purification and characterization of particulate endothelium-derived relaxing factor synthase from cultured and native bovine aortic endothelial cells. Proc. Natl. Acad. Sci. USA.

[36] Baydoun A.R., Emery P.W., Pearson J.D., Mann G.E. (1990). Substrate-dependent regulation of intracellular amino acid concentrations in cultured bovine aortic endothelial cells. Biochem. Biophys. Res. Commun.

[37] Böger R.H., Bode-Böger S.M., Tsao P.S., Lin P.S., Chan J.R., Cooke J.P. (2000). An endogenous inhibitor of nitric oxide synthase regulates endothelial adhesiveness for monocytes. J. Am. Coll. Cardiol.

[38] Surdacki A. (2008). l-arginine analogs—Inactive markers or active agents in atherogenesis?. Cardiovasc. Hematol. Agents Med. Chem.

[39] Berkowitz D.E., White R., Li D., Minhas K.M., Cernetich A., Kim S., Burke S., Shoukas A.A., Nyhan D., Champion H.C. (2003). Arginase reciprocally regulates nitric oxide synthase activity and contributes to endothelial dysfunction in aging blood vessels. Circulation.

[40] Knowles R.G., Palacios M., Palmer R.M., Moncada S. (1989). Formation of nitric oxide from l-arginine in the central nervous system: A transduction mechanism for stimulation of the soluble guanylate cyclase. Proc. Natl. Acad. Sci. USA.

[41] Hecker M., Walsh D.T., Vane J.R. (1991). On the substrate specificity of nitric oxide synthase. FEBS Lett.

[42] Moali C., Boucher J.L., Sari M.A., Stuehr D.J., Mansuy D. (1998). Substrate specificity of NO synthases: Detailed comparison of l-arginine, homo-l-arginine, their *N* omega-hydroxy derivatives, and *N* omega-hydroxynor-l-arginine. Biochemistry.

[43] Davids M., Teerlink T. (2013). Plasma concentrations of arginine and asymmetric dimethylarginine do not reflect their intracellular concentrations in peripheral blood mononuclear cells. Metabolism.

[44] Morris S.M. (2009). Recent advances in arginine metabolism: Roles and regulation of the arginases. Br. J. Pharmacol.

[45] Ryoo S., Gupta G., Benjo A., Lim H.K., Camara A., Sikka G., Sohi J., Santhanam L., Soucy K., Tuday E. (2008). Endothelial arginase II: A novel target for the treatment of atherosclerosis. Circ. Res.

[46] Kim J.H., Bugaj L.J., Oh Y.J., Bivalacqua T.J., Ryoo S., Soucy K.G., Santhanam L., Webb A., Camara A., Sikka G. (2009). Arginase inhibition restores NOS coupling and reverses endothelial dysfunction and vascular stiffness in old rats. J. Appl. Physiol.

[47] Shin W.S., Berkowitz D.E., Ryoo S.W. (2012). Increased arginase II activity contributes to endothelial dysfunction through endothelial nitric oxide synthase uncoupling in aged mice. Exp. Mol. Med.

[48] Scalera F., Borlak J., Beckmann B., Martens-Lobenhoffer J., Thum T., Täger M., Bode-Böger S.M. (2004). Endogenous nitric oxide synthesis inhibitor asymmetric dimethyl l-arginine accelerates endothelial cell senescence. Arterioscler. Thromb. Vasc. Biol.

[49] Shemyakin A., Kövamees O., Rafnsson A., Böhm F., Svenarud P., Settergren M., Jung C., Pernow J. (2012). Arginase inhibition improves endothelial function in patients with coronary artery disease and type 2 diabetes mellitus. Circulation.

[50] Dumont J., Zureik M., Bauters C., Grupposo M.C., Cottel D., Montaye M., Hamon M., Ducimetière P., Amouyel P., Brousseau T. (2007). Association of *OAZ1* gene polymorphisms with subclinical and clinical vascular events. Arterioscler. Thromb. Vasc. Biol.

[51] Pilz S., Meinitzer A., Tomaschitz A., Kienreich K., Dobnig H., Schwarz M., Wagner D., Drechsler C., Piswanger-Sölkner C., März W. (2013). Associations of homoarginine with bone metabolism and density, muscle strength and mortality: Cross-sectional and prospective data from 506 female nursing home patients. Osteoporos. Int.

[52] Schwartz G.J., Muñoz A., Schneider M.F., Mak R.H., Kaskel F., Warady B.A., Furth S.L. (2009). New equations to estimate GFR in children with CKD. J. Am. Soc. Nephrol.

[53] Staples A., LeBlond R., Watkins S., Wong C., Brandt J. (2010). Validation of the revised Schwartz estimating equation in a predominantly non-CKD population. Pediatr. Nephrol.

[54] Martens-Lobenhoffer J., Bode-Böger S.M. (2012). Quantification of l-arginine, asymmetric dimethylarginine and symmetric dimethylarginine in human plasma: A step improvement in precision by stable isotope dilution mass spectrometry. J. Chromatogr. B Analyt. Technol. Biomed. Life Sci.

[55] Martens-Lobenhoffer J., Bode-Böger S.M. (2006). Fast and efficient determination of arginine, symmetric dimethylarginine, and asymmetric dimethylarginine in biological fluids by hydrophilic-interaction liquid chromatography-electrospray tandem mass spectrometry. Clin. Chem.

[56] Martens-Lobenhoffer J., Postel S., Tröger U., Bode-Böger S.M. (2007). Determination of ornithine in human plasma by hydrophilic interaction chromatography-tandem mass spectrometry. J. Chromatogr. B Analyt. Technol. Biomed. Life Sci.

[57] Touboul P.-J., Hennerici M.G., Meairs S., Adams H., Amarenco P., Bornstein N., Csiba L., Desvarieux M., Ebrahim S., Fatar M. (2007). Mannheim carotid intima-media thickness consensus (2004–2006). Cerebrovasc. Dis.

